# Systematic Evaluation of Chromatographic Parameters for Isoquinoline Alkaloids on XB-C18 Core-Shell Column Using Different Mobile Phase Compositions

**DOI:** 10.1155/2018/9624327

**Published:** 2018-02-20

**Authors:** Ireneusz Sowa, Sylwia Zielińska, Jan Sawicki, Anna Bogucka-Kocka, Michał Staniak, Ewa Bartusiak-Szcześniak, Maja Podolska-Fajks, Ryszard Kocjan, Magdalena Wójciak-Kosior

**Affiliations:** ^1^Department of Analytical Chemistry, Medical University of Lublin, Chodźki 4a, 20-093 Lublin, Poland; ^2^Department of Pharmaceutical Biology, Wroclaw Medical University, Borowska 211, 50-556 Wroclaw, Poland; ^3^Department of Biology with Genetics, Medical University of Lublin, Chodźki 4a, 20-093 Lublin, Poland

## Abstract

*Chelidonium majus* L. is a rich source of isoquinoline alkaloids with confirmed anti-inflammatory, choleretic, spasmolytic, antitumor, and antimicrobial activities. However, their chromatographic analysis is difficult because they may exist both in charged and uncharged forms and may result in the irregular peak shape and the decrease in chromatographic system efficacy. In the present work, the separation of main *C. majus* alkaloids was optimized using a new-generation XB-C18 endcapped core-shell column dedicated for analysis of alkaline compounds. The influence of organic modifier concentration, addition of salts, and pH of eluents on chromatographic parameters such as retention, resolution, chromatographic plate numbers, and peak asymmetry was investigated. The results were applied to elaborate the optimal chromatographic system for simultaneous quantification of seven alkaloids from the root, herb, and fruit of *C. majus*.

## 1. Introduction

Isoquinoline alkaloids such as coptisine, allocryptopine, protopine, berberine, chelidonine, sanguinarine, and chelerythrine are the main constituents of *Chelidonium majus* L. responsible for biological properties of the plant. They have analgesic, antispasmodic, antibacterial, antiviral, and antifungal activities. Moreover, they show cytotoxic and antiproliferative effects against various types of cancer cell lines [1–4]. Because of the broad spectrum of action, *C. majus* alkaloids are a subject of interest for pharmacology and toxicology; therefore, the effective analytical methods for their investigation are still being developed. Spectrophotometry [5, 6], capillary electrophoresis [7, 8], and thin-layer chromatography [9–12] have been used for this purpose; however, high-performance liquid chromatography is the most common for qualitative and quantitative analyses of *C. majus* [5, 13–17].

Due to the durability, silica-based RP-18 stationary phases are widely used for HPLC separation of plant extracts [18, 19]; however, RP chromatography of alkaloids is rather difficult because they may exist both as free bases and charged forms. Cationic forms strongly interact with residual silanol groups of the RP-type stationary phase and cause the occurrence of the dual retention mechanism (RP and ion-exchange retention mechanism) and result in the peak tailing, irreproducible retention, and poor system efficiency [20].

Due to the basic character of isoquinoline alkaloids, it would be preferable to conduct chromatographic separation at alkaline pH to avoid their ionization; however, silica-based adsorbents are unstable at this condition [21]. Different approaches may be used to eliminate these problems. Alkaline additives to mobile phases, mostly organic amines such as diethylamine, triethylamine, or dimethyloctylamine, are applied to suppress the ionization of the analyte and as silanol blockers [14, 15]. Addition of anionic ion-pairing reagents, for example, sodium dodecyl sulphate or salts (e.g., ammonium acetate, ammonium formate, and sodium phosphate) is also used to improve the chromatographic separation [5, 13, 17, 22]. On the other hand, the silanol-masking effect may be achieved by additional modification of the sorbent surface, for example, endcapping [23, 24].

XB-C18 sorbent is relatively new column filling with trimethylsilane endcapping and additional isobutyl chains. In the present work, an XB reversed-phase column was used to separate the isoquinoline alkaloids typically found in the *C. majus* extract. The influence of organic modifier concentration, addition of salts, and pH of eluents on chromatographic parameters such as retention, resolution, chromatographic plate numbers, and peak asymmetry was investigated. The results were applied to elaborate the optimal chromatographic system for simultaneous quantification of alkaloids from the root, herb, and fruit of *C. majus*.

## 2. Experimental

### 2.1. Chemicals and Reagents

Alkaloid standards such as protopine (Prot), allocryptopine (All), berberine (Berb), chelidonine (Che), chelerythrine (Chele), and sanguinarine (Sang) were purchased from Sigma (St. Louis, MO) and coptisine (Cop) from ChromaDex (USA). Ammonium acetate, ammonium formate, acetic acid, formic acid, HPLC-grade methanol (MeOH), and acetonitrile (ACN) were from Merck (Darmstadt, Germany). Water was deionized and purified by ULTRAPURE Millipore Direct-QVR 3UV-R (Merck, Darmstadt, Germany).

### 2.2. High-Performance Liquid Chromatography

Chromatography was carried out using a VWR Hitachi Chromaster 600 chromatograph (Merck, Darmstadt, Germany) with a spectrophotometric detector (DAD) and EZChrom Elite software (Merck).

The samples were analyzed on an XB-C18 reversed-phase core-shell column (Kinetex, Phenomenex, Aschaffenburg, Germany) (25 cm × 4.6 mm i.d., 5 *μ*m particle size), at a temperature of 25°C and an eluent flow rate of 1 mL/min.

Chromatograms were recorded in the range of wavelength from 220 to 400 nm. The identity of compounds in plant extracts was confirmed by comparison of retention times and spectra with corresponding standards. Peak homogeneity was established comparing the spectrum recorded at the three peak sections upslope, apex, and downslope with the reference spectrum. Additionally, the chromatographic fractions eluted at the retention time characteristic for the investigated alkaloids were collected using a Foxy R1 fraction collector (Teledyne Isco, Lincoln, USA), and their identity was confirmed by direct injection mass spectrometry (micrOTOF-Q II, Bruker Daltonics, Bremen, Germany) using Compass DataAnalysis software version 4.1.

### 2.3. Sample Preparation

The extraction conditions were based on literature [13]. The root, leaf, and fruit of *C. majus* (1 g) were extracted in ultrasonic bath (3 × 15 min) with 10 mL of methanol acidified with 0.05 M HCl. Subsequently, the extracts were combined, evaporated to dryness, and dissolved in 20 mL of methanol.

## 3. Results and Discussion

Sufficient resolution between neighbouring peaks, symmetric peaks, and narrow peaks are the most important for the optimal chromatographic system. A stationary phase and a mobile phase have a crucial impact on these parameters. In our work, different variants of eluent compositions were tested for their suitability in HPLC of isoquinoline alkaloids on a new-generation XB-C18 endcapped core-shell column. The influence of the three variables: concentration of the organic modifier, salt, and pH on resolution (*R*_*S*_), peak asymmetry (*A*_*S*_), and system efficacy (*N*-theoretical plate numbers) for methanol/water and acetonitrile/water solvent systems was investigated.

### 3.1. Optimization of Chromatographic Condition

The chromatographic parameters were established in the range of 20–40% of acetonitrile (ACN) and 30–40% of methanol (MeOH) in water. Acid (acetic or formic) to obtain appropriate pH (3–5.5) and salt (ammomium acetate or ammonium formate) at the concentration range 5–20 mM were added to tested eluents because blocking and suppressing the ionization of residual silanol groups of the stationary phase were necessary to avoid peak splitting or broadening of basic compounds.

In ACN/water and MeOH/water systems, the amount of organic modifier strongly influence the alkaloid retention. Taking into consideration the resolution, the total separation of investigated compounds was obtained for concentration of 20% ACN in the whole tested pH and salt concentration range. At 25% of ACN, the compound All/Che (at pH ≥ 4) or Che/Cop (at pH ≤ 4) partially coeluted. The exemplary *R*_*s*_ values are given in Table 1.

**Figure 1:**
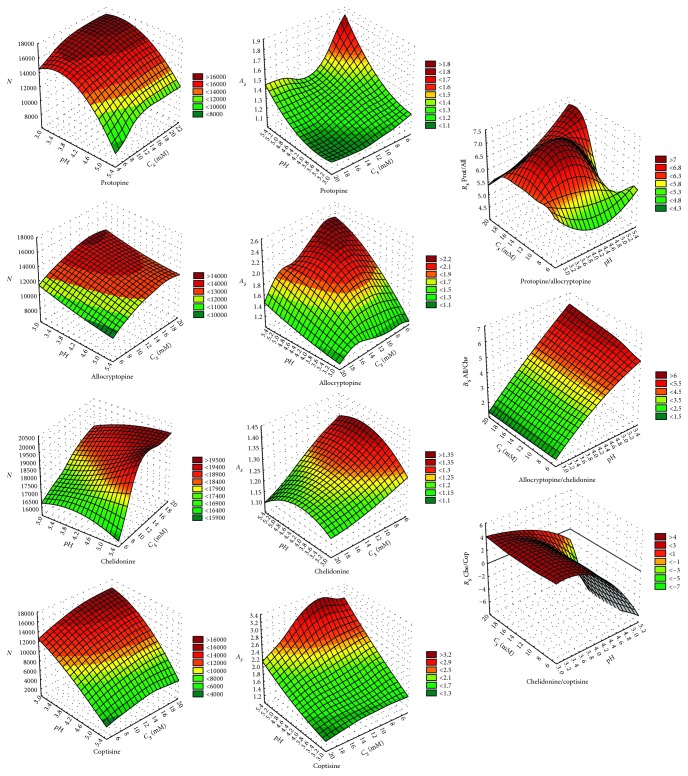
The relationship between theoretical plate numbers (*N*), peak asymmetry (*A*_*s*_), resolution (*R*_*s*_), and pH/ammonium acetate concentration.

In higher concentrations, the majority of compounds were eluted below 10 min (*k* values for protopine, allocryptopine, and chelidonine were lower than 1), and the resolution was poor (Table S1). The concentration of ammonium acetate and pH also affected the alkaloid retention; at lower pH values and at higher salt amounts, retention times were shortened. Moreover, efficiency of the system (*N*), symmetry of peaks (*A*_*s*_), and resolution (*R*_*s*_) strongly depended on these variables. *N*, *A*_*s*_, and *R*_*s*_ values versus pH and salt concentration are presented in Figure 1 and Figure S1.

As can be seen, at pH 5 and at concentration of salt 5 mM, theoretical plate number decreased and peak asymmetry increased significantly, and it resulted in the peak broadening and decreased peak resolution. In contrast to salt concentration, pH had a major impact on *R*_*s*_. Resolution between Che/Cop, Cop/Sang, and Sang/Berb increased at lower pH; in turn, for All/Che, the opposite effect occurred. Moreover, the change of elution order was observed for chelidonine and coptisine at pH 5.

Methanol showed lower elution strength, and the concentration in the range of 30–35% was required to obtain the elution of alkaloids at a reasonable time. Moreover, the order of elution strongly depended on pH and amount of organic modifier and salt (Table 2 and Table S2).

As can be seen, no composition of mobile phases provided sufficient separation within compounds with weaker retention such as protopine, allocryptopine, chelidonine, and coptisine, and all tested chromatographic parameters were worse for methanol/water than for acetonitrile/water eluents.

**Figure 2:**
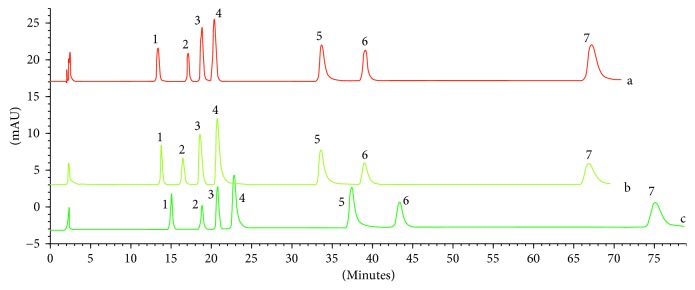
Exemplary chromatograms of the standard mixture obtained at (a) acetonitrile and 10 mM water solution of ammonium formate adjusted to pH 4 with formic acid (20 : 80, v/v), (b) acetonitrile and 20 mM water solution of ammonium acetate adjusted to pH 4 with acetic acid (20 : 80, v/v), and (c) acetonitrile and 10 mM water solution of ammonium acetate adjusted to pH 4 with acetic acid (20 : 80, v/v). (1) protopine, (2) allocryptopine, (3) chelidonine, (4) coptisine, (5) sanguinarine, (6) berberine, and (7) chelerythrine.

In further experiments, ammonium acetate/acetic acid in ACN/water eluents was replaced by ammonium formate/formic acid; however, it had a minor impact on chromatographic parameters. The *R*_*s*_ values and retention times did not differ significantly, and only a slight increase in system efficacy (narrower peaks) was observed. Exemplary chromatograms of the standard mixture obtained at various mobile phase compositions are shown in Figure 2.

**Figure 3:**
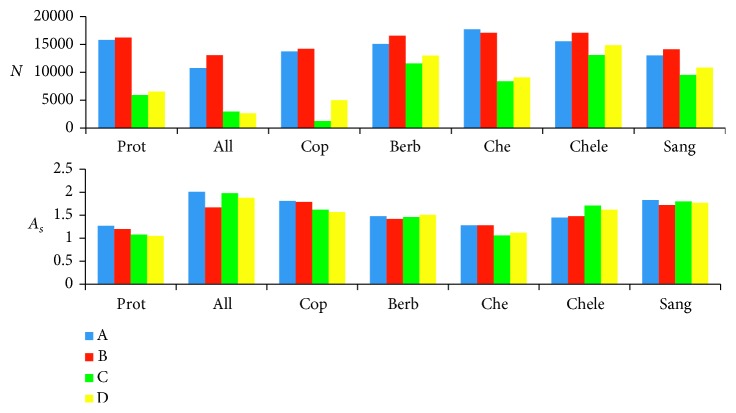
Comparison of theoretical plate numbers and peak asymmetry obtained on XB-C18 column for 20% of acetonitrile and 30% of methanol: (A) acetonitrile and 10 mM water solution of ammonium acetate adjusted to pH 4 with acetic acid; (B) acetonitrile and 10 mM water solution of ammonium formate adjusted to pH 4 with formic acid; (C) methanol and 10 mM water solution of ammonium acetate adjusted to pH 3 with acetic acid; (D) methanol and 10 mM water solution of ammonium acetate adjusted to pH 4 with acetic acid.

The comparison of *N* and *A*_*s*_ for various mobile phase compositions is presented in Figure 3.

Based on the obtained results, acetonitrile at concentration of 20% and water at pH 3-4 with addition of 10–20 mM ammonium acetate or ammonium formate were considered as optimal for isoquinoline alkaloid separation on an XB-C18 core-shell column.

A lot of chromatographic systems for RP separation of isoquinoline alkaloids in *C. majus* were described in literature; however, most of them were more complicated [14, 15, 22] or did not provide sufficient separation for quantitative analysis [25]. Due to additional modification of endcapped octadecyl silica by isobutyl chains in the XB-C18 stationary phase, the interaction of the basic analyte with residual silanol decreased significantly. It allowed to conduct chromatographic separation using milder pH and lower amount of salt addition compared to eluents proposed in literature [13], and it is beneficial for the HPLC system.

### 3.2. Chromatographic Analysis of C. majus Extracts

**Figure 4:**
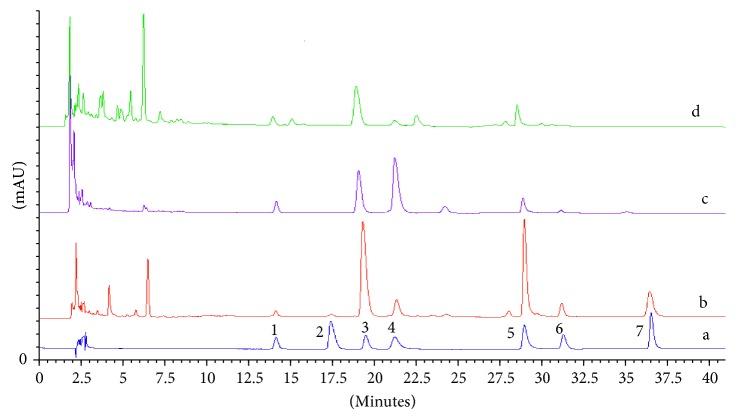
Example of an HPLC-DAD chromatogram of extracts from different parts of *C. majus*: (b) roots, (c) fruits, and (d) herbs and (a) standards of investigated alkaloids: (1) protopine, (2) allocryptopine, (3) chelidonine, (4) coptisine, (5) sanguinarine, (6) berberine, and (7) chelerythrine.

Chromatography of the *C. majus* extract was carried out using the mobile phase consisting of ACN (solvent A) and 10 mM water solution of ammonium acetate adjusted to pH 4 with acetic acid (solvent B) (20 : 80, v/v). High *R*_*s*_ values between coptisine and sanguinarine allowed to use the simple gradient program to shorten the total time of analysis. After 20 min, the elution strength of the mobile phase was increased to accelerate elution of strongly retained sanguinarine, berberine, and chelerythrine. The gradient program was as follows: A 20% and B 80% during 0–20 min, A 25% and B 75% during 20–27 min, and A 30% and B 70% during 27–40 min. The obtained chromatograms are presented in Figure 4.

The data used for identification of the investigated compounds are given in Table 3.

The results of quantitative determination of isoquinoline alkaloids in the root, leaf, and fruit of *C. majus* are given in Table 4, and validation parameters are summarized in Table S3.

## Figures and Tables

**Table 1:** The comparison of retention times and peak resolutions of investigated alkaloids in 20–25% of acetonitrile in water at different pH and ammonium acetate concentrations.

	20% ACN, pH = 3				20% ACN, pH = 4				25% ACN, pH = 3				25% ACN, pH = 4			
	20 mM		10 mM		20 mM		10 mM		20 mM		10 mM		20 mM		10 mM	
	*t*_*R*_	*R*_*S*_	*t*_*R*_	*R*_*S*_	*t*_*R*_	*R*_*S*_	*t*_*R*_	*R*_*S*_	*t*_*R*_	*R*_*S*_	*t*_*R*_	*R*_*S*_	*t*_*R*_	*R*_*S*_	*t*_*R*_	*R*_*S*_
Protopine	10.94	5.38	11.49	6.08	13.16	5.79	13.91	7.25	6.54	4.07	6.66	4.07	7.75	4.20	7.72	4.55
Allocryptopine	13.40	1.36	14.17	1.45	16.81	3.47	17.06	3.35	7.61	1.25	7.73	1.43	8.89	3.98	9.03	3.23
Chelidonine	14.07	4.11	14.90	3.88	18.79	2.68	19.29	3.05	7.97	2.56	8.17	2.60	10.07	1.21	10.07	1.29
Coptisine	16.02	13.65	16.84	14.26	20.35	14.18	21.03	14.95	8.73	8.72	8.92	9.41	10.5	9.57	10.55	9.08
Sanguinarine	24.58	5.62	26.39	5.45	33.71	4.59	37.27	4.07	11.76	5.65	12.11	5.70	14.8	5.38	14.78	5.11
Berberine	28.96	17.00	31.01	17.22	39.12	16.82	42.27	18.23	14.01	12.67	14.43	12.79	17.58	13.25	17.51	12.62
Chelerythrine	47.54	—	51.73	—	66.91	—	74.71	—	20.45	—	21.26	—	26.45	—	26.28	—

**Table 2:** The comparison of retention times and peak resolutions of investigated alkaloids in 35% of methanol in water at different pH and ammonium acetate concentrations.

	pH = 3				pH = 4			
	20 mM		10 mM		20 mM		10 mM	
	*t*_*R*_	*R*_*S*_	*t*_*R*_	*R*_*S*_	*t*_*R*_	*R*_*S*_	*t*_*R*_	*R*_*S*_
Protopine	8.05	0.63	8.34	0.77	10.67	1.12	10.51	1.10
Allocryptopine	8.47	0.96	8.92	0.76	11.56	2.25	11.23	2.36
Chelidonine	9.11	1.18	9.49	1.18	13.37	0.08	13.15	0.30
Coptisine	9.68	10.60	10.18	10.02	13.43	10.48	13.37	12.94
Berberine	15.88	2.57	17.23	2.75	23.82	7.93	23.9	14.47
Sanguinarine	17.73	13.12	19.47	13.26	36.85	6.21	48.31	5.71
Chelerythrine	29.85	—	33.4	—	50.16	—	63.02	—

**Table 3:** The data used for identification of the investigated compounds.

	Retention time (min)	UV-Vis spectrum	*m/z*
Protopine	13.91	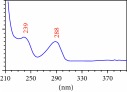	354.135
Allocryptopine	17.06	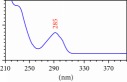	369.157
Chelidonine	19.29	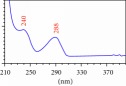	353.126
Coptisine	20.86	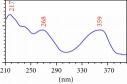	320.092
Sanguinarine	28.25	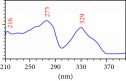	332.092
Berberine	30.68	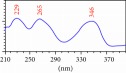	336.123
Chelerythrine	35.71	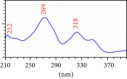	348.123

**Table 4:** The content of investigated alkaloids (mg/100 g ± SD) in different parts of *C. majus*.

	Root	Herb	Fruit
Protopine	15.7 ± 1.1	20.2 ± 1.6	22.4 ± 2.0
Allocryptopine	11.4 ± 0.8	—	—
Chelidonine	140.1 ± 10.4	65.2 ± 5.1	57.4 ± 5.1
Coptisine	50.1 ± 4.1	20.8 ± 1.5	247.2 ± 20.9
Sanguinarine	311.6 ± 22.4	29.8 ± 2.1	20.1 ± 1.9
Berberine	52.7 ± 3.9	0.8 ± 0.1	10.4 ± 0.9
Chelerythrine	100.3 ± 7.8	<LOQ	<LOQ

## Supplementary Materials

Supplementary Materials Table S1: the comparison of retention times and peak resolutions of investigated alkaloids in 30% of acetonitrile in water at different pH and ammonium acetate concentration. Table S2: the comparison of retention times and peak resolutions of investigated alkaloids in 30% of methanol in water at different pH and ammonium acetate concentration. Table S3: calibration data for quantification of investigated alkaloids. Figure S1: the relationship between theoretical plate numbers (N), peak asymmetry (As), resolution (Rs) and pH/ammonium acetate concentration.
