# Supplementary material for: Systematic Evaluation of Chromatographic Parameters for Isoquinoline Alkaloids on XB-C18 Core-Shell Column Using Different Mobile Phase Compositions
Source: J Anal Methods Chem. 2018 Feb 20;2018:9624327. doi: 10.1155/2018/9624327 (PMC5838492; doi:10.1155/2018/9624327)
Supplement: Supplementary Materials — Table S1: the comparison of retention times and peak resolutions of investigated alkaloids in 30% of acetonitrile in water at different pH and ammonium acetate concentration. Table S2: the comparison of retention times and peak resolutions of investigated alkaloids in 30% of methanol in water at different pH and ammonium acetate concentration. Table S3: calibration data for quantification of investigated alkaloids. Figure S1: the relationship between theoretical plate numbers (N), peak asymmetry (As), resolution (Rs) and pH/ammonium acetate concentration. [file 9624327.f1.docx]

Table S1. The comparison of retention times and peak resolutions of investigated alkaloids in 30% of acetonitrile in water at different pH and ammonium acetate concentration.

| 30 % ACN pH=3 | | | | | 30% ACN pH4 | | | |
| --- | --- | --- | --- | --- | --- | --- | --- | --- |
|  | 20 mM | | 10 mM | | 20 mM | | 10 mM | |
|  | t_R_ | R_S_ | t_R_ | R_S_ | t_R_ | R_S_ | t_R_ | R_S_ |
| protopine | 4.67 | 3.30  1.13  1.87  5.81  5.13  8.78 | 4.69 | 3.19  1.48  1.80  5.68  5.11  8.95 | 5.3 | 2.95  3.21  0.73  6.07  4.40  8.85 | 5.48 | 3.54  3.25  0.83  6.83  5.12  9.75 |
| allocryptopine | 5.23 |  | 5.23 |  | 5.90 |  | 6.17 |  |
| chelidonine | 5.44 |  | 5.48 |  | 6.58 |  | 6.83 |  |
| coptisine | 5.82 |  | 5.83 |  | 6.74 |  | 7.02 |  |
| sanguinarine | 7.10 |  | 7.13 |  | 8.49 |  | 8.93 |  |
| berberine | 8.36 |  | 8.43 |  | 10.02 |  | 10.58 |  |
| chelerythrine | 11.04 |  | 11.16 |  | 13.47 |  | 14.38 |  |

Table S2. The comparison of retention times and peak resolutions of investigated alkaloids in 30% of methanol in water at different pH and ammonium acetate concentration

|  | 30% MeOH, pH=3 | | | | | 30% MeOH, pH=4 | | | | | |
| --- | --- | --- | --- | --- | --- | --- | --- | --- | --- | --- | --- |
|  | 20 mM | | 10 mM | | |  | 20 mM | |  | 10 mM | |
|  | t_R_ | R_S_ |  | t_R_ | R_S_ |  | t_R_ | R_S_ |  | t_R_ | R_S_ |
| protopine | 12.56 | 0.01  1.55  0.89  11.27  2.67  17.92 | protopine | 13.37 | 2.07  0.87  2.84  14.33  2.88  17.40 | protopine | 17.352 | 0.45  1.70  1.24  18.81  1.92  11.44 | protopine | 18.12 | 3.33  1.41  2.93  18.17  7.49  11.07 |
| allocryptopine | 12.57 |  | allokryptopine | 15.16 |  | allokryptopine | 18.369 |  | allocryptopine | 21.25 |  |
| coptisine | 13.94 |  | chelidonine | 15.92 |  | coptizine | 21.698 |  | chelidonine | 23.19 |  |
| chelidonine | 14.87 |  | coptizine | 18.28 |  | chelidonine | 25.279 |  | coptisine | 26.92 |  |
| sanguinarine | 30.25 |  | berberine | 33.95 |  | sanguinarine | 51.425 |  | berberine | 52.17 |  |
| berberine | 33.48 |  | sanguinarine | 37.88 |  | berberine | 56.916 |  | sanguinarine | 73.63 |  |
| chelerythrine | 62.33 |  | chelerythrine | 72.23 |  | chelerythrine | 105.961 |  | chelerythrine | 121.1 |  |

Table S3. Calibration data for quantification of investigated alkaloids.

|  | Linear regression  equation | Correlation  coefficient (r) | Precision (% relative  standard deviation, RSD) |
| --- | --- | --- | --- |
| protopine | y=574259x+45655 | 0.9985 | 1,014 - 1,411% |
| coptisine | y=900425x+64533 | 0.9991 | 1.334 – 2.361% |
| allocryptopine | y=500291x+54538 | 0.9983 | 0.971 – 1.421% |
| chelidonine | y=628233x+63692 | 0.9999 | 1.024 – 2.514% |
| berberine | y=662383x+69768 | 0.9992 | 1.081 – 1.555% |
| sanguinarine | y=525974x+55045 | 0.9981 | 1.002 – 1.894% |
| chelerythrine | y=497552x+56545 | 0.9998 | 0.994 – 2.014% |


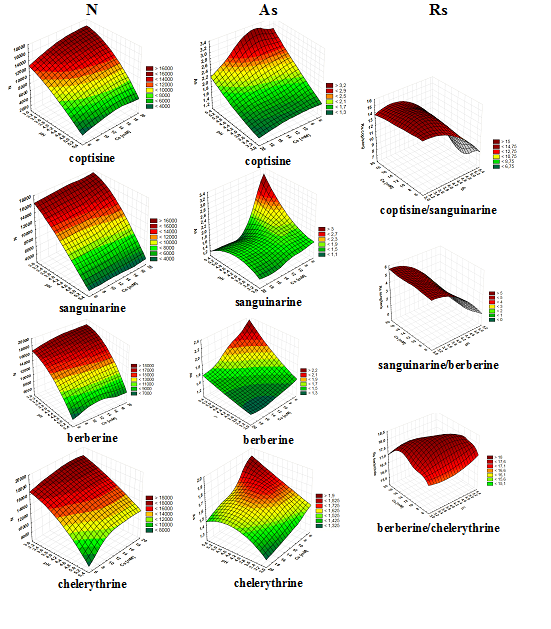


Fig S1. The relationship between theoretical plate numbers (N), peak asymmetry (As), resolution (Rs) and pH/ammonium acetate concentration.
